# Supplementary material for: AdiY acts as a cytoplasmic pH sensor via histidine protonation to regulate acid stress adaptation in Escherichia coli
Source: J Bacteriol. 2025 Dec 23;208(1):e00542-25. doi: 10.1128/jb.00542-25 (PMC12826058; doi:10.1128/jb.00542-25)
Supplement: Figure S1 — Functional analysis of AdiY histidine variants. [file jb.00542-25-s0001.pdf]

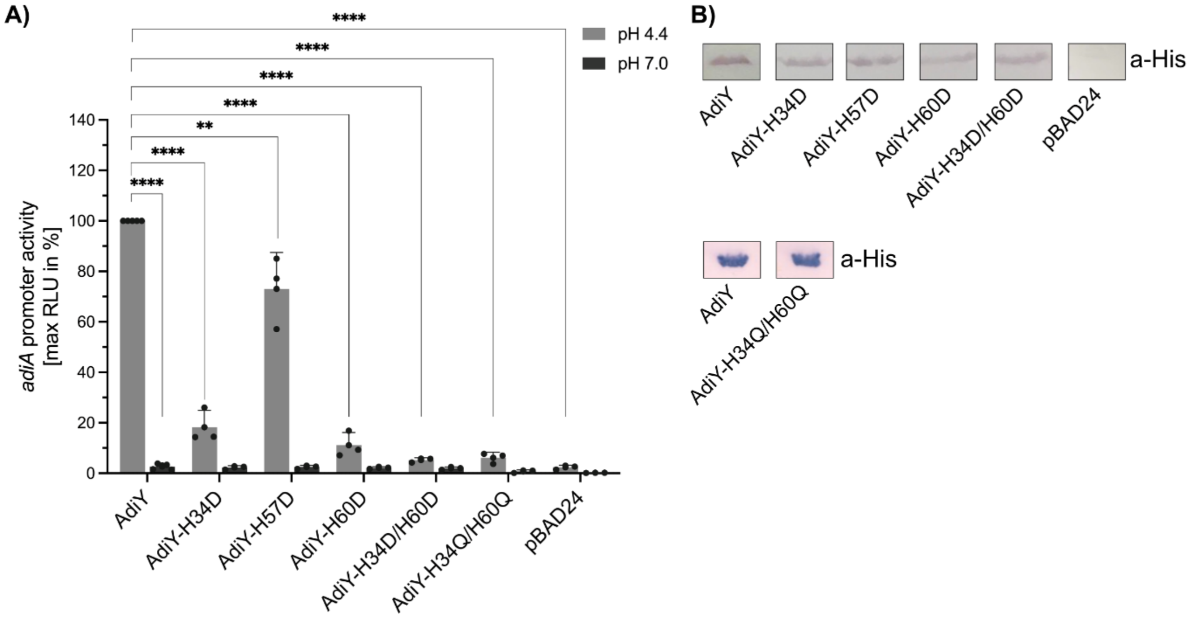

Figure S1. Functional analysis of AdiY histidine variants. *E. coli* MG1655 Δ*adiY* was co-transformed with the reporter plasmid pBBR1-MCS5-P_{adiA}-*lux* and plasmid-based AdiY wild-type or variants. Cells were grown in a citrate-buffered medium adjusted to pH 4.4 (grey) or pH 7.0 (dark grey) and supplemented with 0.1% L-arabinose. Data are reported as relative light units (RLUs) in counts per second per milliliter per OD_{600}, and the maximal RLU after 2 h of growth is shown. All experiments were performed at least three times ($n \geq 3$), and error bars represent the standard deviation of the means. Statistics: Student's unpaired two-sided t test; ** $p = 0.044$; **** $p < 0.0001$. (B) Production of AdiY wild-type or AdiY variants was confirmed by western blot analysis using antibodies against the His-tag. Protein bands correspond to a 29-kDa protein. The blot is shown split because the samples were initially loaded onto the gel in a different order. The bands were cut and rearranged to achieve the desired order in the graph.
