# Supplementary material for: AdiY acts as a cytoplasmic pH sensor via histidine protonation to regulate acid stress adaptation in Escherichia coli
Source: J Bacteriol. 2025 Dec 23;208(1):e00542-25. doi: 10.1128/jb.00542-25 (PMC12826058; doi:10.1128/jb.00542-25)
Supplement: Figure S2 — Production and purification of wild-type AdiY and the AdiY-H34A/H60A variant. [file jb.00542-25-s0002.pdf]

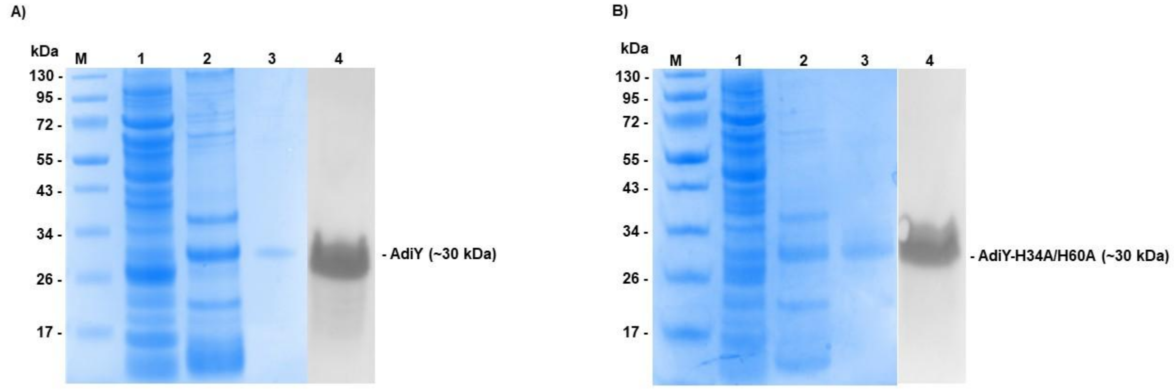

Figure S2: Production and purification of wild-type AdiY and the AdiY-H34A/H60A variant. (A) SDS-PAGE analysis of AdiY. Lane M, molecular weight marker; lane 1, whole-cell lysate after induction; lane 2, eluate from Ni^{2+}-affinity chromatography; lane 3, purified protein after size-exclusion chromatography; lane 4, confirmation through western blot, using anti-His antibodies, confirming production and purification of the His_6-tagged proteins. **(B)** SDS-PAGE analysis of AdiY-H34A/H60A variant. Lane M, molecular weight marker; lane 1, whole-cell lysate after induction; lane 2, eluate from Ni^{2+}-affinity chromatography; lane 3, purified protein after size-exclusion chromatography; lane 4, confirmation through western blot, using anti-His antibodies confirming production and purification of the His_6-tagged proteins.
