# Supplementary material for: AdiY acts as a cytoplasmic pH sensor via histidine protonation to regulate acid stress adaptation in Escherichia coli
Source: J Bacteriol. 2025 Dec 23;208(1):e00542-25. doi: 10.1128/jb.00542-25 (PMC12826058; doi:10.1128/jb.00542-25)
Supplement: Figure S3 — pH-dependent binding of AdiY wild-type variant with target promoters analyzed by surface plasmon resonance (SPR) spectroscopy. [file jb.00542-25-s0003.pdf]

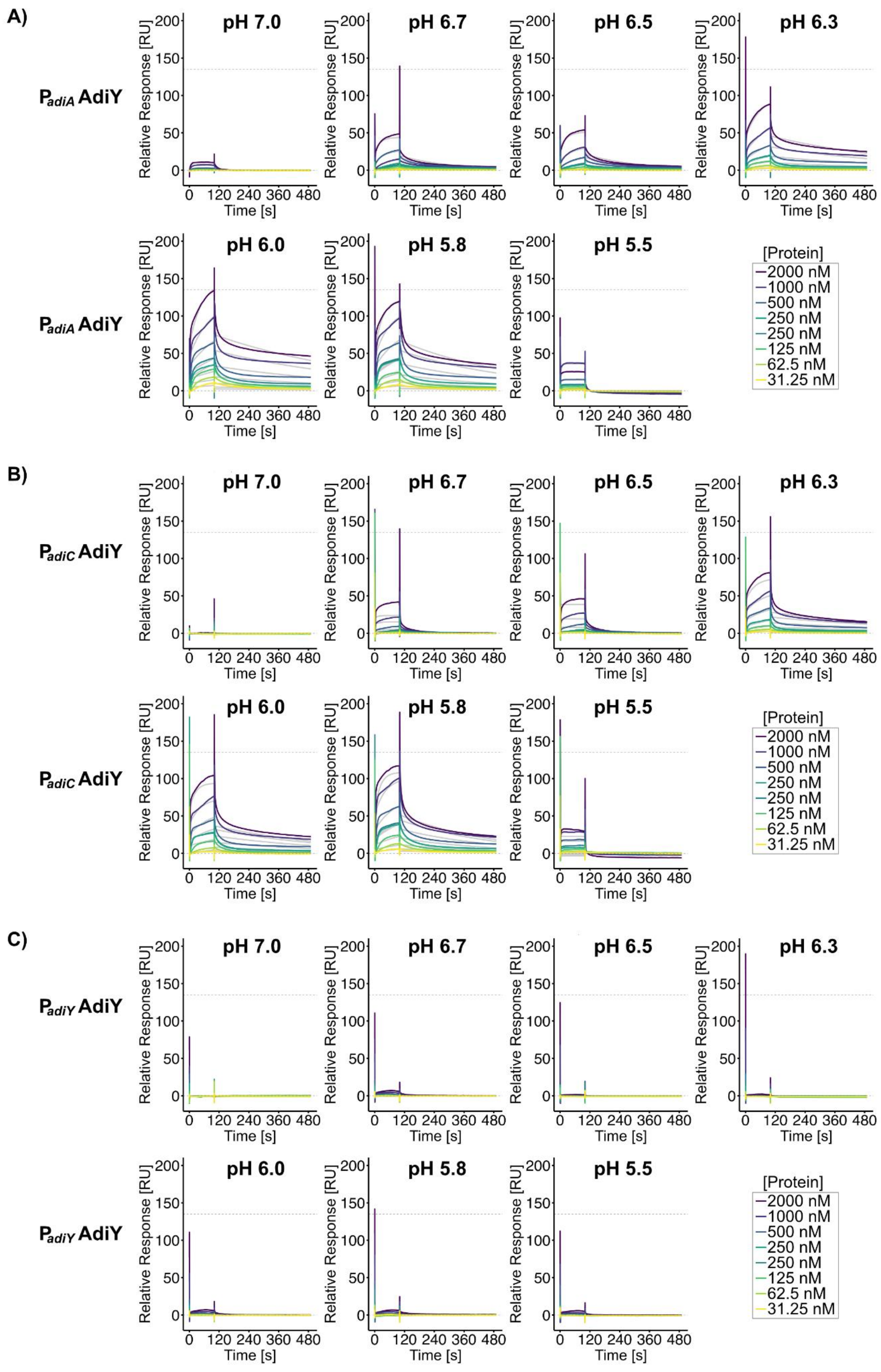

Figure S3: pH-dependent binding of AdiY wild-type variant with target promoters analyzed by surface plasmon resonance (SPR) spectroscopy. Biotinylated DNA fragments comprising the promoters of *adiA* (P_{adiA}) **(A)**, *adiC* (P_{adiC}) **(B)** or *adiY* (P_{adiY})

(C) were captured on SA sensor chips. Solutions of purified AdiY wild-type were passed over the sensor chip at different pH values ranging from 7.0 to 5.5. Sensorgram color coding corresponds to increasing protein concentrations, as indicated in the panel on the right. The dashed line at 0 RU represents the baseline, while the dashed line at 135 RU marks the maximal response of wild-type AdiY to the *adiA* promoter at pH 6.0. Where applicable, association rate (k_a) and dissociation (k_d) rate constants and the equilibrium dissociation constants (K_D) are summarized in Table 1.
