# Supplementary material for: AdiY acts as a cytoplasmic pH sensor via histidine protonation to regulate acid stress adaptation in Escherichia coli
Source: J Bacteriol. 2025 Dec 23;208(1):e00542-25. doi: 10.1128/jb.00542-25 (PMC12826058; doi:10.1128/jb.00542-25)
Supplement: Figure S4 — pH-dependent binding of the AdiY-H34A/H60A variant with target promoters analyzed by surface plasmon resonance (SPR) spectroscopy. [file jb.00542-25-s0004.pdf]

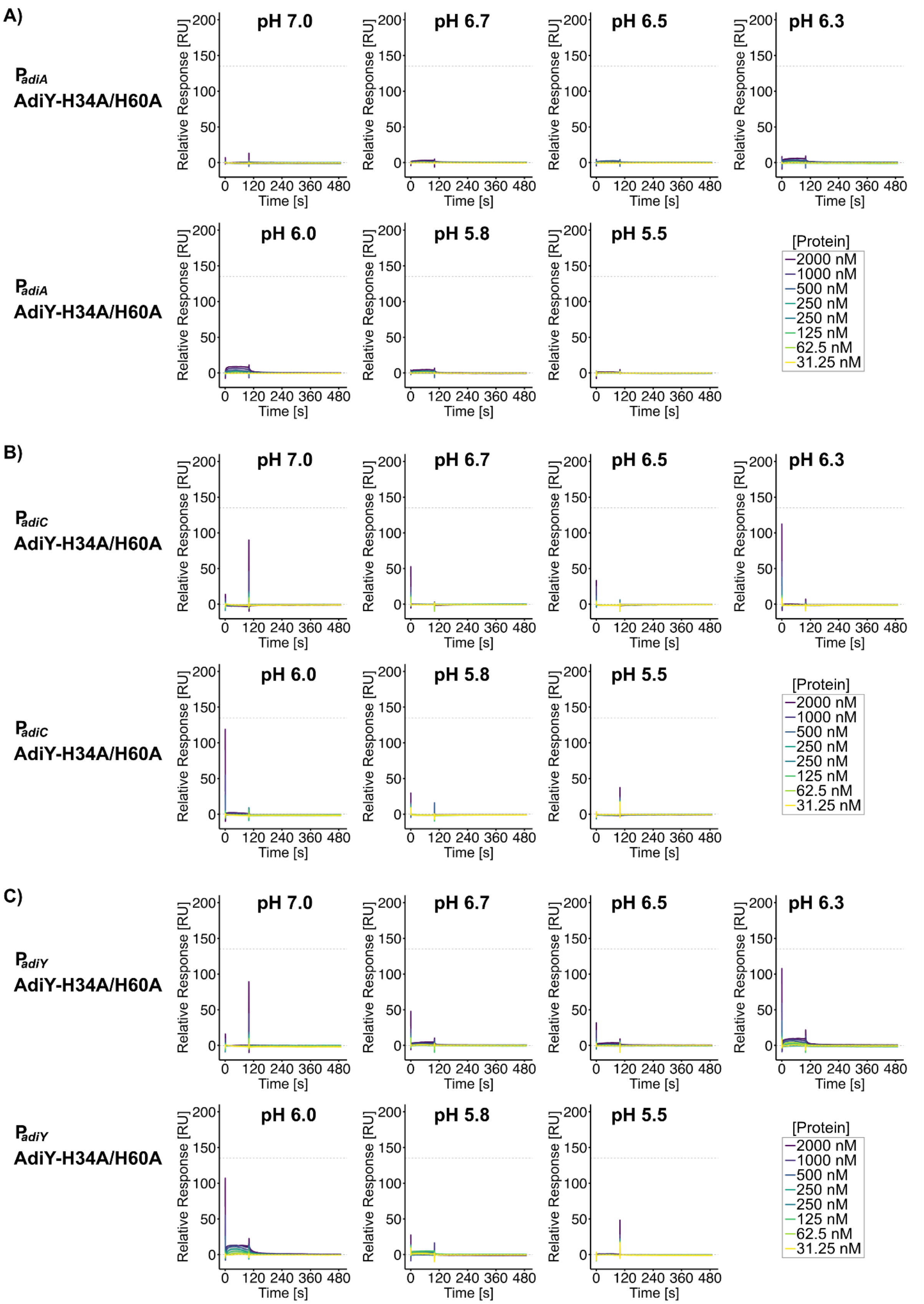

Figure S4: pH-dependent binding of the AdiY-H34A/H60A variant with target promoters analyzed by surface plasmon resonance (SPR) spectroscopy. Biotinylated DNA fragments comprising the promoters of *adiA* (P_{adiA}) **(A)**, *adiC* (P_{adiC})

(B) or *adiY* (P_{adiY}) **(C)** were captured on SA sensor chips. Solutions of the purified AdiY-H34A/H60A variant were passed over the sensor chip at different pH values ranging from 7.0 to 5.5. Sensorgram color coding corresponds to increasing protein concentrations, as indicated in the panel on the right. The dashed line at 0 RU represents the baseline, while the dashed line at 135 RU marks the maximal response of wild-type AdiY to the *adiA* promoter at pH 6.0 (Figure S3).
