# Supplementary material for: AdiY acts as a cytoplasmic pH sensor via histidine protonation to regulate acid stress adaptation in Escherichia coli
Source: J Bacteriol. 2025 Dec 23;208(1):e00542-25. doi: 10.1128/jb.00542-25 (PMC12826058; doi:10.1128/jb.00542-25)
Supplement: Figure S5 — Alignment of 17 AdiY homologs of representative species. [file jb.00542-25-s0005.pdf]

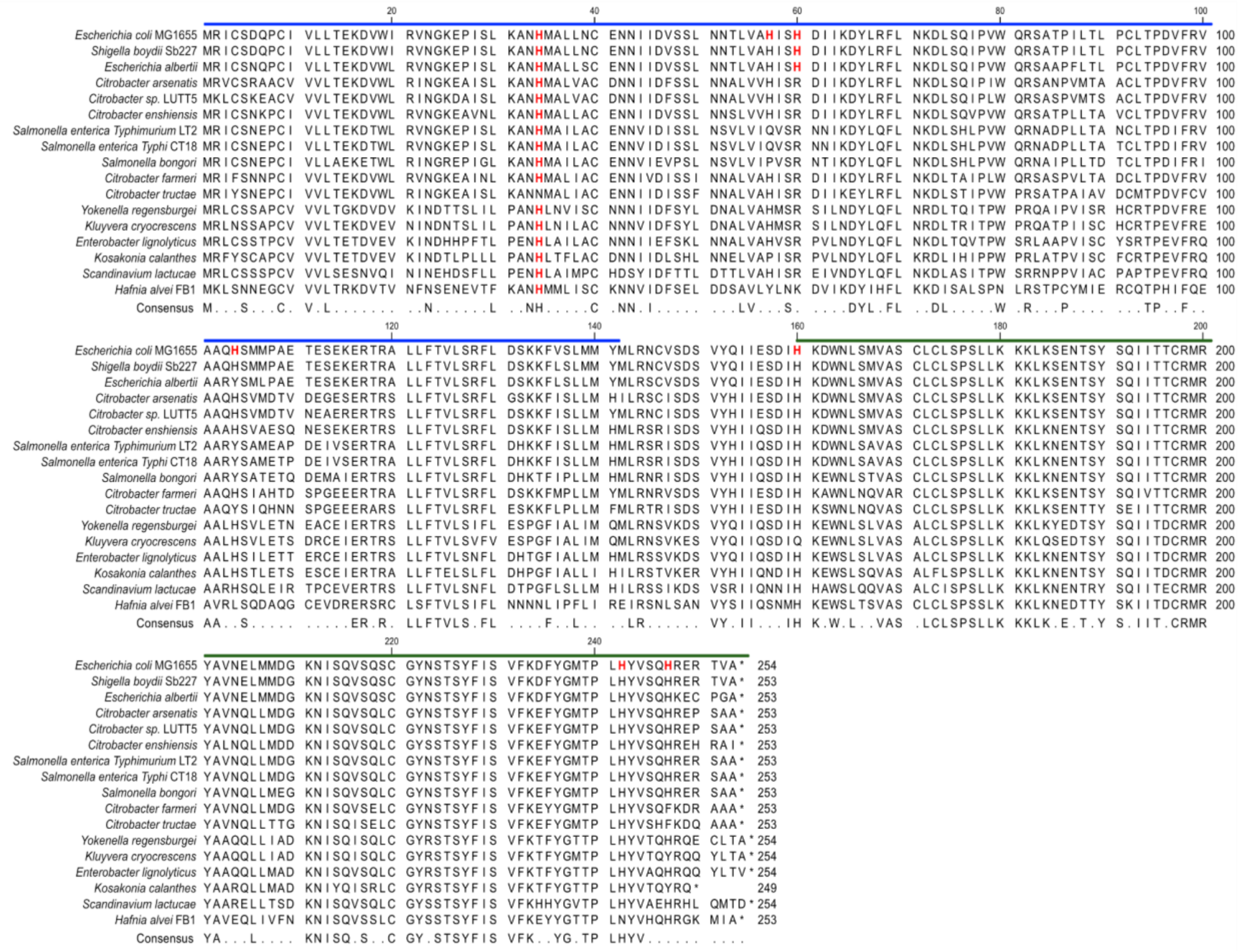

Figure S5: Alignment of 17 AdiY homologs of representative species. Seventeen non-redundant AdiY orthologs were aligned using a progressive algorithm (CLC Main Workbench v24.0.1; gap-open 10, gap-extension 1). The *E. coli* K-12 MG1655 AdiY (UniProt P33234) is shown on the top line and provides the reference numbering indicated above the alignment. Species names are listed at left, and stop codons are marked with an asterisk. The consensus line reports positions with ≥90% conservation across sequences (letters) and variable positions (dots). Domain boundaries are indicated above the alignment: N-terminal domain (blue) and C-terminal domain (green). Histidine residues are highlighted in red. Accession numbers for all sequences are provided in Table S2.
