# Supplementary material for: AdiY acts as a cytoplasmic pH sensor via histidine protonation to regulate acid stress adaptation in Escherichia coli
Source: J Bacteriol. 2025 Dec 23;208(1):e00542-25. doi: 10.1128/jb.00542-25 (PMC12826058; doi:10.1128/jb.00542-25)
Supplement: Figure S6 — AlphaFold 3 structural predictions of AdiY and its interaction with DNA. [file jb.00542-25-s0006.pdf]

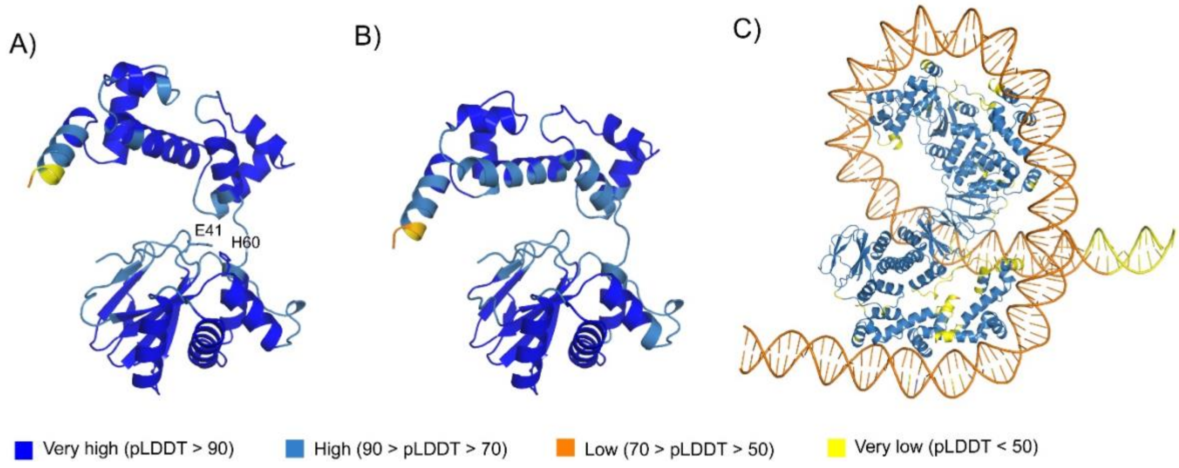

Figure S6: AlphaFold 3 structural predictions of AdiY and its interaction with DNA. (A) Predicted structure of wild-type AdiY. Residue-wise prediction confidence (pLDDT) is color coded (blue: very high (pLDDT>90), cyan: high (pLDDT>70), orange: low (pLDDT>50), yellow: very low (pLDDT<50)), showing very high confidence across most secondary structure elements, with reduced reliability limited to flexible loops. (B) Predicted structure of the AdiY-H34A/H60A mutant. The AraC-family fold is preserved, and the model exhibits high to very high confidence in structured regions, with low-confidence scores confined to loop regions (using the same color scale as in A). (C) Predicted AdiY–DNA complex obtained with AlphaFold 3 multimer mode. Protein regions interacting with DNA are predicted with high confidence, whereas peripheral DNA segments and flexible loops show lower reliability (color scale as in A).
