# Supplementary material for: AdiY acts as a cytoplasmic pH sensor via histidine protonation to regulate acid stress adaptation in Escherichia coli
Source: J Bacteriol. 2025 Dec 23;208(1):e00542-25. doi: 10.1128/jb.00542-25 (PMC12826058; doi:10.1128/jb.00542-25)
Supplement: Table S1 — Kinetic parameters of AdiY binding to its target promoter determined by surface plasmon resonance (SPR). [file jb.00542-25-s0007.pdf]

Table S1: Kinetic parameters of AdiY binding to its target promoter determined by surface plasmon resonance (SPR). Association rate (k_a) and dissociation (k_d) rate constants, and the equilibrium dissociation constants (K_D) were determined for AdiY binding to the *adiA* and *adiC* promoter fragments at the indicated pH values. Values represent means from two (pH 5.8, 6.3, and 6.5) or three (pH 6.0) independent experiments (N), with standard deviations (SD). Measurements were performed using increasing concentrations of His_6-AdiY (31.25 nM to 2000 nM) passed over a DNA-coated sensor chip. Binding to the *adiY* promoter was not detectable and is therefore not included in the table. Stoichiometry refers to DNA:protein ratios.

		Association constant (k_a) [M^{-1} s^{-1}]		Dissociation constant (k_d) [s^{-1}]		Equilibrium constant (K_D) [M]			
Pro-moter	pH	Mean	SD	Mean	SD	Mean	SD	N	Stoichi-ometry
adiA	5.8	1.32×10^4	2.83×10^3	1.73×10^3	5.44×10^4	1.29×10^{-7}	1.36×10^{-8}	2	1:4
adiA	6.0	1.01×10^4	1.53×10^3	1.70×10^3	1.15×10^4	1.70×10^{-7}	2.32×10^{-8}	3	1:4
adiA	6.3	7.02×10^3	1.63×10^3	2.35×10^3	7.64×10^4	3.57×10^{-7}	1.92×10^{-7}	2	1:3
adiA	6.5	1.54×10^4	1.13×10^3	7.50×10^3	4.11×10^3	4.98×10^{-7}	3.03×10^{-7}	2	1:2
adiC	5.8	2.83×10^4	9.55×10^3	6.00×10^3	4.76×10^3	1.95×10^{-7}	1.02×10^{-7}	2	1:4
adiC	6.0	1.60×10^4	5.72×10^3	1.80×10^3	2.46×10^4	1.20×10^{-7}	3.44×10^{-8}	3	1:4
adiC	6.3	1.37×10^4	1.13×10^3	1.99×10^3	6.43×10^4	8.99×10^{-7}	1.10×10^{-6}	2	1:3
adiC	6.5	4.03×10^3	2.36×10^3	1.78×10^2	3.61×10^3	59.1×10^{-7}	3.78×10^{-6}	2	1:2
