# Supplementary material for: AdiY acts as a cytoplasmic pH sensor via histidine protonation to regulate acid stress adaptation in Escherichia coli
Source: J Bacteriol. 2025 Dec 23;208(1):e00542-25. doi: 10.1128/jb.00542-25 (PMC12826058; doi:10.1128/jb.00542-25)
Supplement: TABLE S2 — Accession numbers of AdiY homologs and corresponding organism names. [file jb.00542-25-s0008.pdf]

Name	Accession number of AdiY homolog
Escherichia coli MG1655	NP_418540.1
Shigella boydii Sb227	WP_001217059.1
Escherichia albertii	WP_059224522.1
Citrobacter arsenatis	WP_133087188.1
Citrobacter sp. LUTT5	WP_044702149.1
Citrobacter enshiensis	WP_301702143.1
Salmonella enterica Typhimurium LT2	NP_463160.1
Salmonella enterica Typhi CT18	WP_001217076.1
Salmonella bongori	WP_001217070.1
Citrobacter farmeri	WP_042321229.1
Citrobacter tructae	WP_436858142.1
Yokenella regensburgei	WP_120816530.1
Kluyvera cryocrescens	WP_061279769.1
Enterobacter lignolyticus	WP_013368007.1
Kosakonia calanthes	WP_342322099.1
Scandinavium lactucae	WP_319786484.1
Hafnia alvei FB1	WP_025799565.1

Table S2: Accession numbers of AdiY homologs and corresponding organism names. AdiY homologs were identified using BLASTp against the NCBI RefSeq Select protein database (downloaded September 2025). The query sequence was the AdiY *from Escherichia coli* K-12 MG1655.
